# Head-to-Head Comparison of Magic Leap 2 and HoloLens 2 Using Patient-Specific Phantoms in Orthopedic Oncology: A Feasibility Study

**DOI:** 10.3390/s26216966

**Published:** 2026-11-04

**Authors:** Naqash Nasir, Laura Cercenelli, Stefano Stradiotti, Michele Fiore, Marta Bortoli, Fabrizio Ferretti, Roy Eagleson, Brent A. Lanting, Achille Tarsitano, Massimiliano De Paolis, Emanuela Marcelli

**Affiliations:** 1 Orthopedics and Traumatology Department, IRCCS Azienda Ospedaliero-Universitaria di Bologna, 40138 Bologna, Italy; michele.fiore9@unibo.it (M.F.); marta.bortoli@outlook.com (M.B.); massimiliano.depaolis@aosp.bo.it (M.D.P.); 2 eDIMES Lab-Laboratory of Bioengineering, Department of Medical and Surgical Sciences, University of Bologna, 40138 Bologna, Italy; stefano.stradiotti@outlook.com (S.S.); fabrizio.ferretti9@unibo.it (F.F.); emanuela.marcelli@unibo.it (E.M.); 3 Biomedical Engineering, Faculty of Engineering, Western University, London, ON N6A 3K7, Canada; eagleson@uwo.ca; 4 Division of Orthopaedic Surgery, Department of Surgery, London Health Sciences Centre, University Hospital, London, ON N6A 5A5, Canada; brent.lanting@lhsc.on.ca; 5 Oral and Maxillofacial Surgery Unit, IRCCS Azienda Ospedaliero-Universitaria di Bologna, 40138 Bologna, Italy; achille.tarsitano2@unibo.it; 6 Maxillofacial Surgery Unit, Department of Biomedical and Neuromotor Sciences, University of Bologna, 40126 Bologna, Italy; 7 Health Sciences and Technologies, Interdepartmental Center for Industrial Research (CIRI-SDV), University of Bologna, 40124 Bologna, Italy; 8 IRCCS Azienda Ospedaliero-Universitaria di Bologna, 40138 Bologna, Italy

**Keywords:** augmented reality, mixed reality, orthopedic oncology, surgery, tracking, head-mounted display, 3D printing

## Abstract

Augmented reality (AR) represents an emerging technology that is increasingly being integrated into surgical navigation across various medical disciplines, including orthopedic surgery. This preclinical study investigated the application of AR in orthopedic oncology and performed a comparative assessment of two head-mounted displays (HMDs): Microsoft HoloLens 2 and Magic Leap 2. The study evaluated the feasibility of AR-assisted tracing of planned osteotomies for bone tumor resections in orthopedic surgery. The objective was to evaluate the visualization capabilities, spatial guidance performance, and user interaction provided by the two AR platforms under controlled preclinical conditions. Accuracy of AR-guided tracing of planned osteotomies was quantified using custom cutting guides incorporating grooves of 1 mm, 2 mm, and 3 mm to measure deviations from the planned osteotomy lines. Both AR HMDs were tested under identical experimental conditions, and their performance and accuracy were comparatively analyzed. Magic Leap 2 demonstrated a trend toward greater accuracy in AR-guided tracing of osteotomies, achieving 82%, 95%, and 100% accuracy within 1 mm, 2 mm, and 3 mm thresholds of the planned osteotomy lines, respectively. In comparison, the HoloLens 2 achieved 65%, 85%, and 95% accuracy within the same respective thresholds. Ergonomic assessment also suggested favorable user comfort and handling characteristics for Magic Leap 2. Further studies involving larger cohorts and comparisons with established navigation techniques are required to confirm the potential advantages of HMD-based AR guidance for bone tumor resections and other orthopedic procedures.

## 1. Introduction

Augmented reality (AR) is an innovative technology that merges computer-generated virtual images with the real-world environment, enabling an interactive and immersive experience [1]. By using devices such as smartphones, tablets, and more recently head-mounted displays (HMDs), AR enhances visual perception by embedding or overlaying virtual elements onto the user’s natural field of view [2].

With its growing adoption in the medical domain, this technology is now extensively utilized for various applications ranging from education and surgical training to preoperative planning and intraoperative navigation in the operating room. By providing an intuitive visualization of complex three-dimensional (3D) anatomy and structures, AR technology has been widely adopted by various surgical fields. Examples include neurosurgery [3], urological surgery [4,5,6], cardiovascular surgery [7], spinal surgery [8], breast surgery [9], gastrointestinal and hepatic surgery [10,11], orthopedic surgery, cranio-maxillofacial surgery [12,13,14], and dentistry [15].

In the field of orthopedic oncology, the risk of positive surgical margins and subsequent recurrence has long been a concern among surgeons [16]. In order to mitigate this issue, meticulous preoperative planning is essential to define optimal surgical margins, thereby ensuring complete tumor excision in cases of bone malignancies.

To date, several studies have highlighted the application of AR in orthopedic oncology, employing various HMDs to enhance surgical precision. Molina et al. utilized the Augmedics system to assist in osteotomy and pedicle screw placement in a patient diagnosed with lumbar chondroma [8]. García-Sevilla et al. underscored the utility of AR for pelvic tumor resections using the Microsoft HoloLens 2 [17]. Similarly, Moreta-Martínez et al. assessed the accuracy of the HoloLens 1 in a patient-specific phantom model of Ewing’s sarcoma [18]. Furthermore, Pose-Díez-de-la-Lastra conducted a comparative analysis between HoloLens 1 and HoloLens 2, evaluating their performance in tumor resections involving both upper and lower extremities [2].

This study aimed to evaluate and compare the accuracy of two prominent HMDs used in AR: HoloLens 2 (Microsoft) and Magic Leap 2 (Magic Leap Inc.), utilizing patient-specific phantoms in orthopedic oncology. While HoloLens 2 has been extensively studied and applied across various surgical disciplines [13,19,20], this study aimed to assess whether the newer-generation device, Magic Leap 2, offers improved accuracy for surgical guidance. The experimental design assessed the accuracy achieved when using AR overlays from both devices to guide surgical tasks on a pelvic oncology phantom by comparing the accuracy of AR-guided marking of planned resection lines obtained with each device. In order to determine which equipment was deemed more appropriate for intraoperative use, an ergonomic and usability survey was also administered to the participants.

## 2. Materials and Methods

### 2.1. Patient Selection and Clinical Data

The study included a 48-year-old female patient diagnosed with chondrosarcoma involving the iliac region, at the Department of Orthopedics, IRCCS Azienda Ospedaliero-Universitaria di Bologna. The tumor measured 9.1 cm × 2.0 cm × 5.5 cm. To ensure patient confidentiality, a unique case identifier was assigned. Informed consent was obtained from the participant prior to data collection, in accordance with the ethical principles outlined in the Declaration of Helsinki (1975, revised in 2013). The study protocol received approval from the Research Ethics Committee of the hospital.

### 2.2. Virtual Content Creation and Phantom Development

The process of three-dimensional (3D) printing for phantom development started with acquisition of patient’s CT (Computed Tomography) images for segmentation. The slice thickness of all CT scans was 0.5 mm and segmentations were performed using Mimics Core Medical 28.0 software (Materialise, Leuven, Belgium). After CT segmentation, 3D virtual models of the pelvic region were imported into 3-Matic 20.0 software (Materialise, Leuven, Belgium), a computer-aided design (CAD) platform used to generate the surgical resection planes (and corresponding lines), as well as the templates for accuracy evaluation.

**Figure 1:**
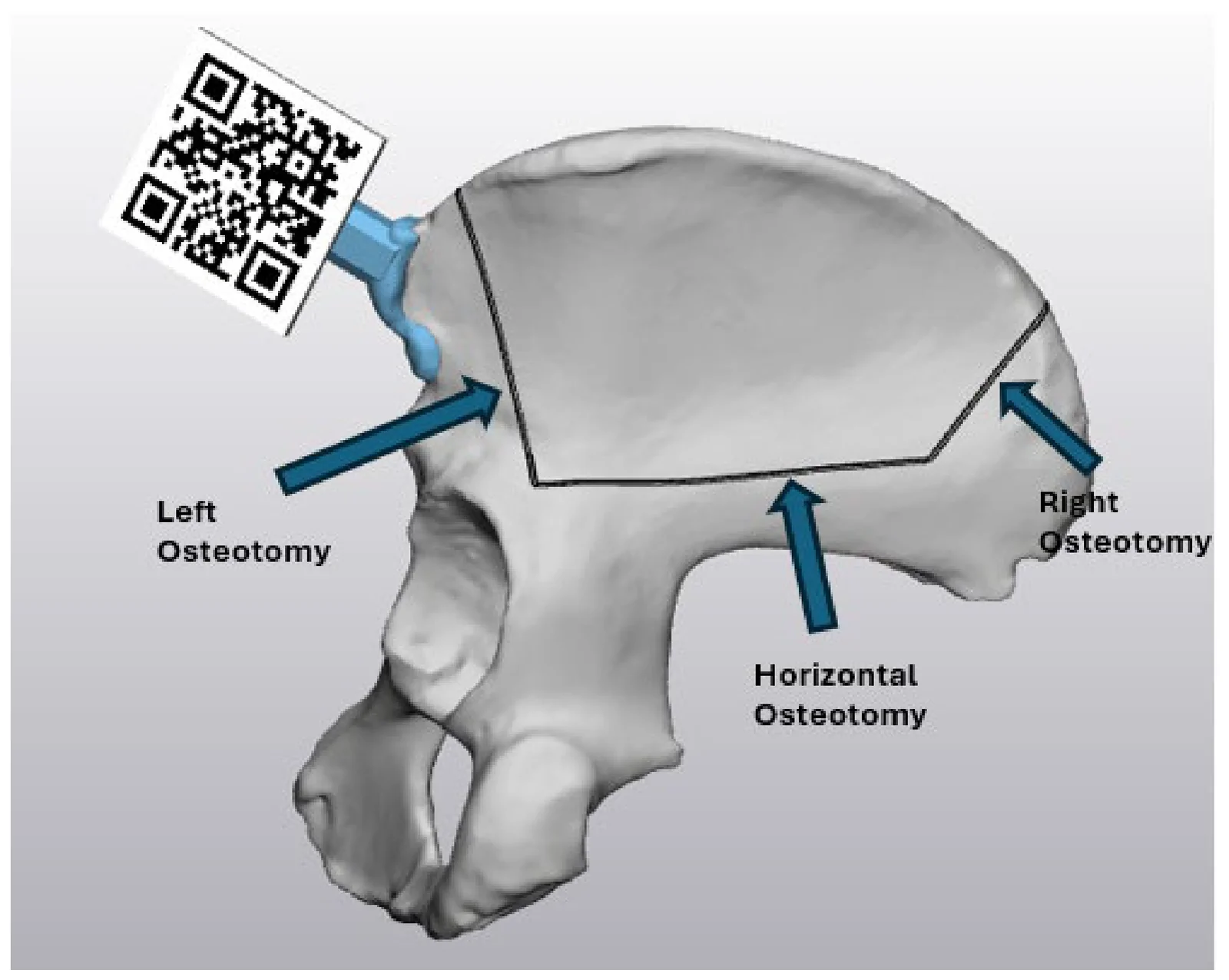
Three osteotomy planes: right, left and horizontal for iliac tumor.

A marker-based QR code system was selected for tracking and registration, leveraging the stable reference provided by the hard bone structures (Figure 1). The QR code marker was designed with dimensions of 5 cm × 5 cm and fabricated using PolyJet printing technology (J720 printer, Stratasys, Minnetonka, MN, United States).

A holder was used to affix the AR tracking marker on the pre-defined bony area. The surgeons determined the marker’s position by considering numerous factors of the surgical procedure, including patient positioning, viewing angle, and line of sight, ensuring that the marker remained visible throughout the procedure without obstructing the surgical workflow (Figure 1). Following the CAD and surgical planning, tumorous bones and accuracy templates were printed with photopolymeric white resin using a Formlabs 3 stereolithography printer (Formlabs Inc., Somerville, MA, USA). Since these phantoms and patient-specific instruments were intended solely for in-vitro research, they were neither manufactured using biocompatible resin nor sterilized.

### 2.3. Development of AR Application

The AR application was developed using Unity (version 2022.3.9f1) and programmed in C#, ensuring compatibility with both Magic Leap 2 and HoloLens 2. Vuforia Engine 10.20 (PTC Inc., Boston, MA, USA) was utilized to enable a marker-based tracking approach (“Image Target” tracking).

This is a Vuforia Engine tracking method based on the recognition and tracking of a pre-defined image (Image Target) that provides sufficient details to be detected, typically a QR-code pattern. The Vuforia Engine detects and tracks the image by comparing extracted natural features from the camera image against the known Image Target previously reviewed in terms of tracking performance and stored in a cloud database. The Image Target can be downloaded from the database as a package suitable for integration in the Unity app. The QR marker was positioned in a precise and reproducible location relative to the patient’s anatomy using the 3D-printed patient-specific holder. This allowed for projection of virtual models onto corresponding physical structures using pre-calculated registration coordinates. No dedicated quantitative measurements of registration error, tracking jitter, drift, or system latency were performed as part of the present protocol. The AR application was deployed on HoloLens 2 (via Universal Windows Platform build, minimum platform version 10.0.10240.0) and Magic Leap 2 (via Android-based build). Device-specific SDKs were used to support spatial tracking and rendering capabilities while maintaining an identical application workflow across platforms. The application was developed to operate at the native display refresh rate of the AR headset.

Prior to the evaluation of each headset, the manufacturer-recommended calibration procedure was performed to adjust the display to each participant’s interpupillary distance, ensuring optimal visualization conditions and consistency across devices.

The AR interface featured interactive elements, such as the native bone, the tumor and its outline, and the planned resection lines, which could be selectively visualized by the surgeon through gesture-based interaction with the HMD menu.

The application projected resection lines directly onto the phantom, offering real-time visual guidance to ensure precise adherence to the intended trajectory. This approach illustrates the desired clinical advantage of AR in oncological resection, where precise margin delineation is critical to lowering the likelihood of positive margins and local recurrence.

### 2.4. Evaluation of AR System

The implementation of AR in surgical disciplines requires rigorous evaluation to establish its feasibility, reliability, and precision in replicating complex surgical maneuvers. In orthopedic oncology, accuracy is paramount to ensure that osteotomies align with preoperative plans, thereby minimizing complications and enhancing surgical outcomes. To assess the performance of the developed AR system, we designed an in-vitro study employing the above-described patient-specific pelvic phantom and 3D-printed templates. This methodology enabled controlled evaluation of AR-guided tracing of osteotomies in terms of reproducibility and accuracy.

Three osteotomy planes (right, left, and horizontal) were defined for the iliac tumor (Figure 1). These planes represented the critical resection lines required to perform oncologic resections or corrective osteotomies with precision. Each planned cut was subsequently superimposed onto the model using both devices’ AR views to provide reference guidance for task execution.

**Figure 2:**
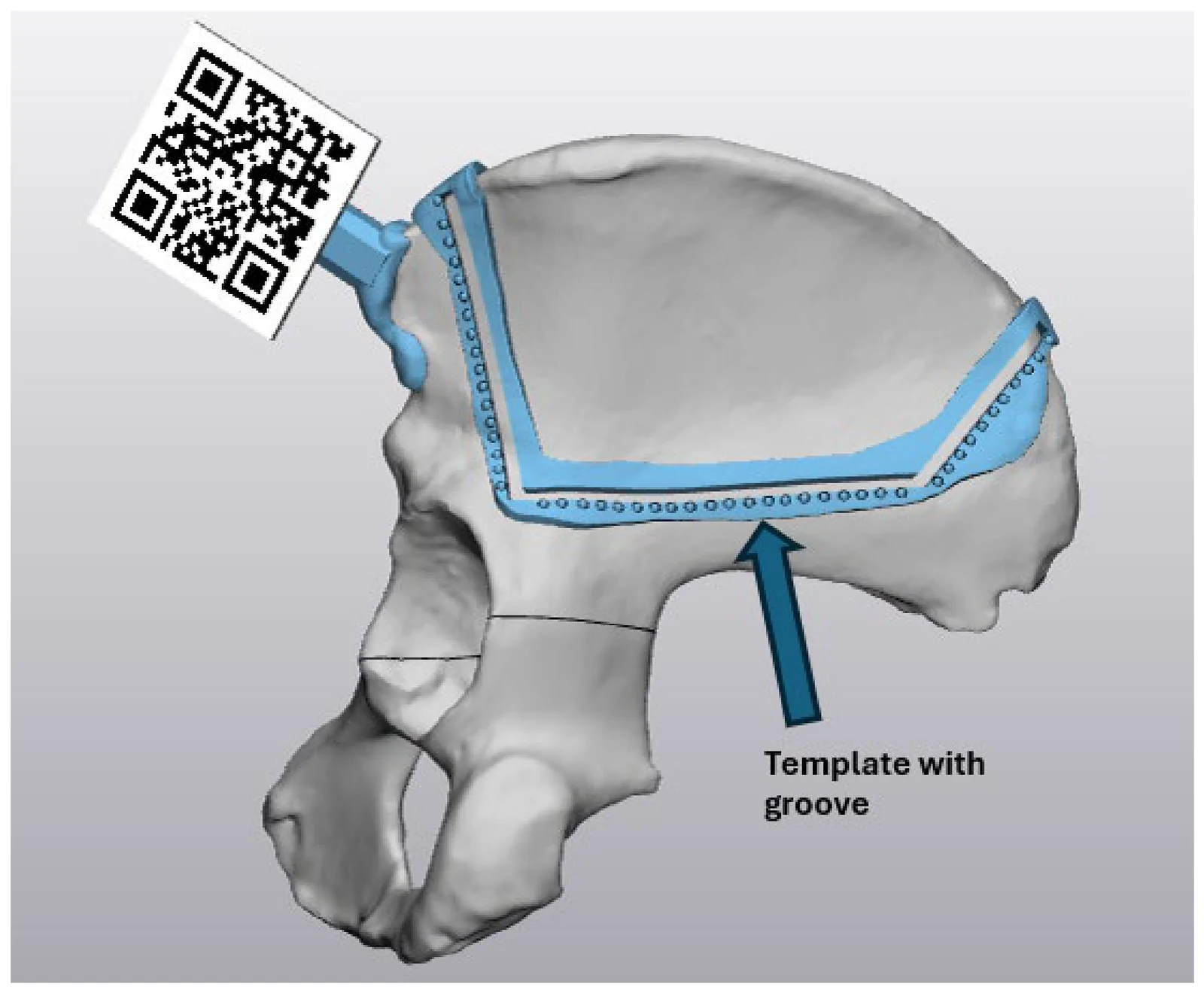
Grooved template with predefined reference points (circles) used to assess accuracy.

To validate the accuracy of AR-guided marking of planned resection lines, a grooved template was designed (Figure 2). This template incorporated predefined reference points along the osteotomy planes, serving as a benchmark for assessing the deviation of the AR-guided tracing from the ideal trajectory.

**Figure 3:**
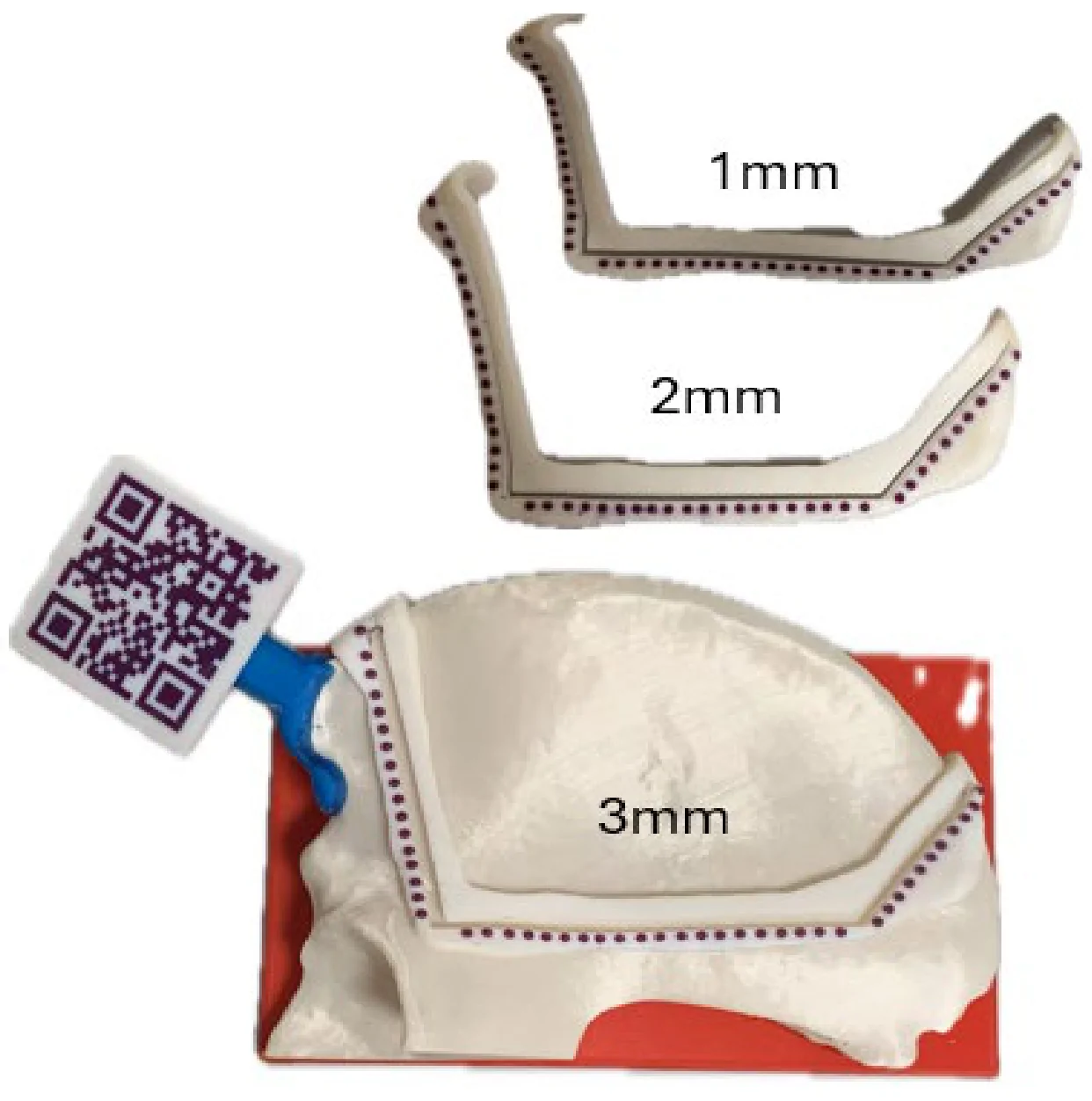
Template grooves with varying widths (1 mm, 2 mm and 3 mm).

The validation templates were 3D-printed, with grooves of varying widths: 1 mm, 2 mm, and 3 mm (Figure 3). The selected thresholds were chosen to reflect clinically relevant tolerances for oncologic bone tumor resections in orthopedic surgery; however, they do not capture the full distribution of geometric deviations. The templates provided a reproducible and objective tool for quantifying three accuracy levels of osteotomy line marking by assessing the deviation between the planned osteotomy trajectories and the lines traced by the surgeons on the model.

The groove-based validation templates were manufactured directly from the digital design using a standardized 3D-printing workflow. Prior to testing, all templates underwent visual inspection and fitting verification on the corresponding anatomical model to confirm proper seating and alignment with the planned osteotomy planes.

Accuracy was defined as the percentage of sampled trajectory points located within the acceptable tolerance region represented by the groove of the validation template. Traced trajectories were resampled at 1.5 mm intervals using the predefined reference points distributed along the osteotomy planes and incorporated into the validation template. Each sampled point was classified as either inside (score = 1) or outside (score = 0) the groove boundaries. Accuracy was computed as the ratio of points classified as inside the slot to the total number of sampled points (expressed as a percentage).

### 2.5. Experimental Protocol

The accuracy of AR-guided marking of resection lines was evaluated using the two AR devices (HoloLens 2 and Magic Leap 2).

Ten participants were enrolled, including five orthopedic surgeons and five engineers, to reflect the multidisciplinary context in which AR technologies are developed and applied in surgical practice. As this was a preliminary preclinical evaluation of the hardware platforms, both clinical and technical stakeholders were considered relevant end-users. Participants had comparable baseline familiarity with AR systems, assessed through a preliminary screening questionnaire.

Each of the 10 participants performed three tracing trials with each AR headset to ensure consistent conditions and reliable data collection, resulting in 30 trials per device and a total of 60 tracing trials across the study.

**Figure 4:**
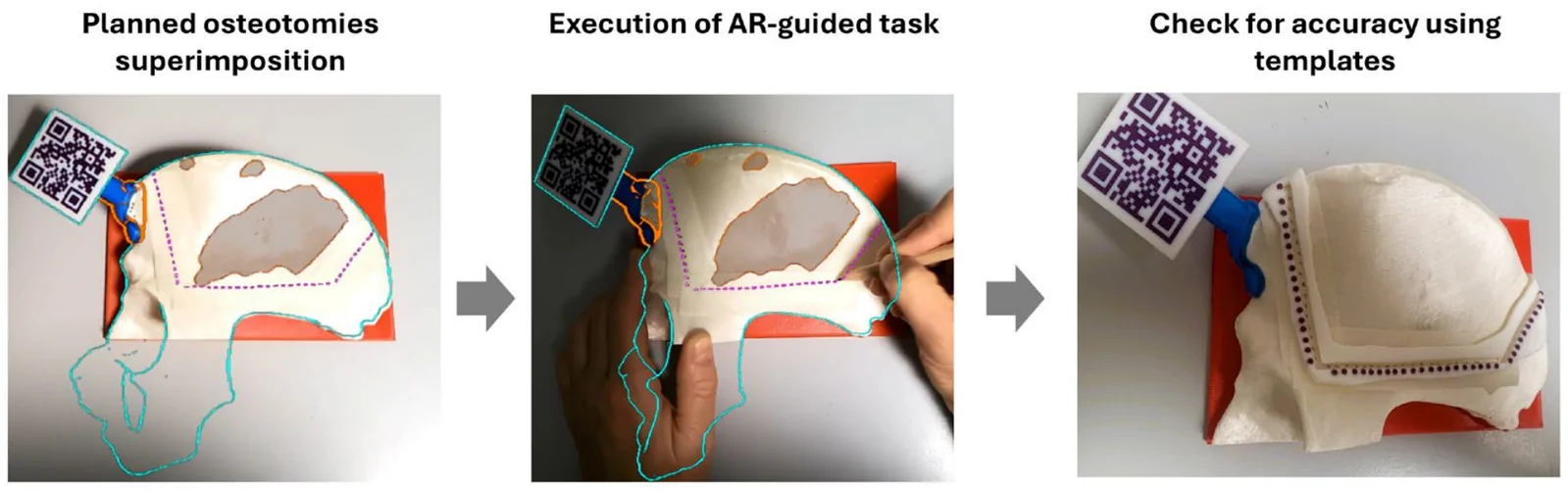
Workflow of performing AR-assisted tracing of the planned osteotomies (dashed fuchsia lines).

The experimental protocol was structured into three sequential stages (Figure 4):

1. Superimposition of Planned Osteotomies

The QR code marker was fixed to the pelvic phantom, allowing precise registration between the digital plan and the physical phantom. The preoperatively defined osteotomy lines were projected onto the phantom using both AR headsets.

2. Execution of AR-Guided Tracing

Participants performed the marking of the osteotomy lines while wearing the AR headsets, aligning their marking pencil with the holographically projected cutting lines. To mitigate potential learning effects, the order of headset use was counterbalanced across participants. Specifically, the first five participants used HoloLens2 followed by MagicLeap2, whereas the subsequent five participants performed the tasks in the reverse order. The AR overlay provided continuous visual feedback, guiding the trajectory and orientation of each osteotomy. The brightness of the virtual lines could be adjusted, for example dimmed, to enhance visibility of the real environment and improve observation of the tracking instrument relative to the projected line.

3. Accuracy Assessment

Following the completion of the line-tracing tasks along the planned osteotomy line, the model was fitted with the grooved templates. Line-tracing deviations from the grooves were documented, with the tolerance levels (1 mm, 2 mm, 3 mm) serving as thresholds for quantifying the accuracy achieved.

**Figure 5:**
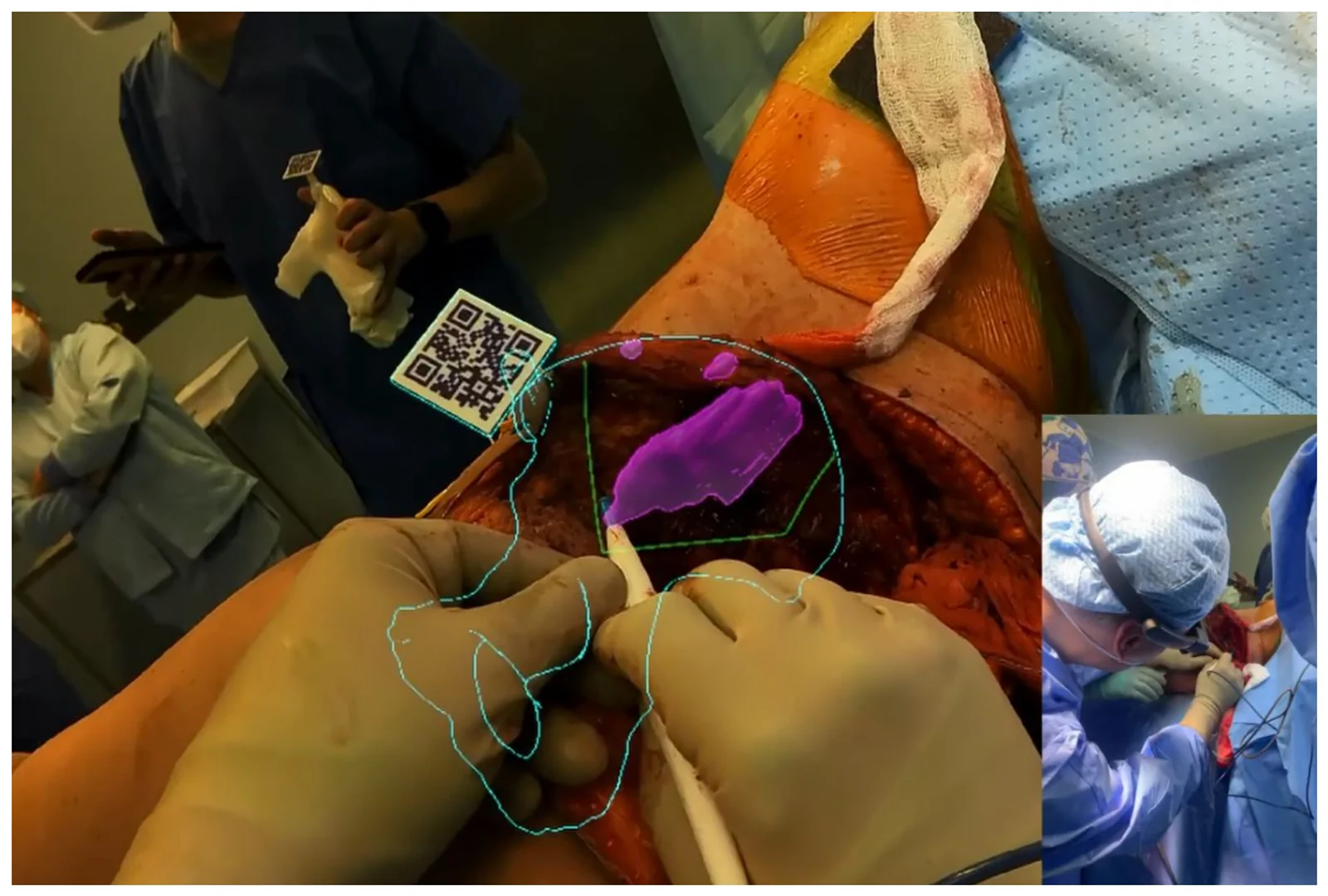
Intraoperative use of Magic Leap 2 demonstrating AR-guided visualization of a pelvic tumor (in fuchsia) and resection lines (in green), with holographic overlay aligned to the surgical field (in cyan).

The feasibility of Magic Leap 2 was further assessed in the operating room environment, focusing on its practical usability, ergonomics, and integration within the standard surgical workflow (Figure 5). This operating room demonstration was not designed as a comparative evaluation between Magic Leap 2 and HoloLens 2. Rather, it served as a feasibility assessment to explore the integration of AR HMD technology into a real surgical workflow and to evaluate its practical usability in a realistic clinical environment.

### 2.6. Technical Comparison Between Magic Leap 2 and HoloLens 2

The key technical features and differences between the Magic Leap 2 and HoloLens 2 headsets are presented in Table 1 to provide a comparative overview.

### 2.7. Ergonomics and Usability Survey Comparison Between HoloLens 2 and Magic Leap 2

The ergonomic design of these devices is crucial for their practical adoption in the OR, especially during extended use in high-pressure settings. Components including comfort, fit, weight distribution, interaction quality, and fatigue can profoundly influence user performance, satisfaction, and long-term usage.

The survey was conducted with 10 participants to assess critical ergonomic characteristics pertinent to clinical and engineering tasks, utilizing subjective user feedback to identify strengths and problems related to both devices. User responses were quantified using a Likert scale, providing insights into how well these devices align with user-centered design principles. The results highlight the significance of ergonomics in the advancement of AR technology and provide guidance for subsequent versions or future work on these devices.

The survey consisted of a comprehensive investigation into AR-assisted processes, concentrating on users with clinical expertise. The analysis demonstrates uniform patterns in user preferences, influencing device choices in clinical contexts.

A 5-point Likert scale (1 = worse, 5 = best) was employed to rate ergonomic and usability attributes. Nine core questions targeted device-specific factors. The questionnaire items related to these factors are tabulated as follows (Table 2). As the questionnaire was developed in-house and was not formally validated, the ergonomics assessment was considered exploratory and analyzed descriptively.

### 2.8. Statistical Analysis

Paired *t*-tests were conducted to compare the resulting accuracy between HoloLens 2 (HL2) and Magic Leap 2 (ML2) for each osteotomy site and accuracy level. The effect size was estimated using Cohen’s *d* for paired samples. The participant was considered the statistical unit of analysis. For each headset, the three repetitions performed by each participant were averaged to obtain a single representative value per outcome measure. Statistical analyses were subsequently performed on participant-level data. Prior to inferential testing, normality assumptions were assessed using the Shapiro–Wilk test. Data analyses were conducted with Stata 19/SE software (StataCorp, College Station, TX, USA). To account for multiple comparisons, Bonferroni correction was applied separately within each predefined task grouping (Left, Bottom, Right, and ALL), considering the three accuracy thresholds (1, 2, and 3 mm) as a family of simultaneous comparisons. Accordingly, statistical significance was set at *p* < 0.0167 (0.05/3).

## 3. Results

### 3.1. Accuracy of AR-Guided Marking of Planned Osteotomies

The comparative evaluation of AR-assisted oncological resections (osteotomy lines) using the HL2 and ML2 demonstrated progressive improvements in accuracy across varying thresholds (1 mm, 2 mm, and 3 mm).

**Figure 6:**
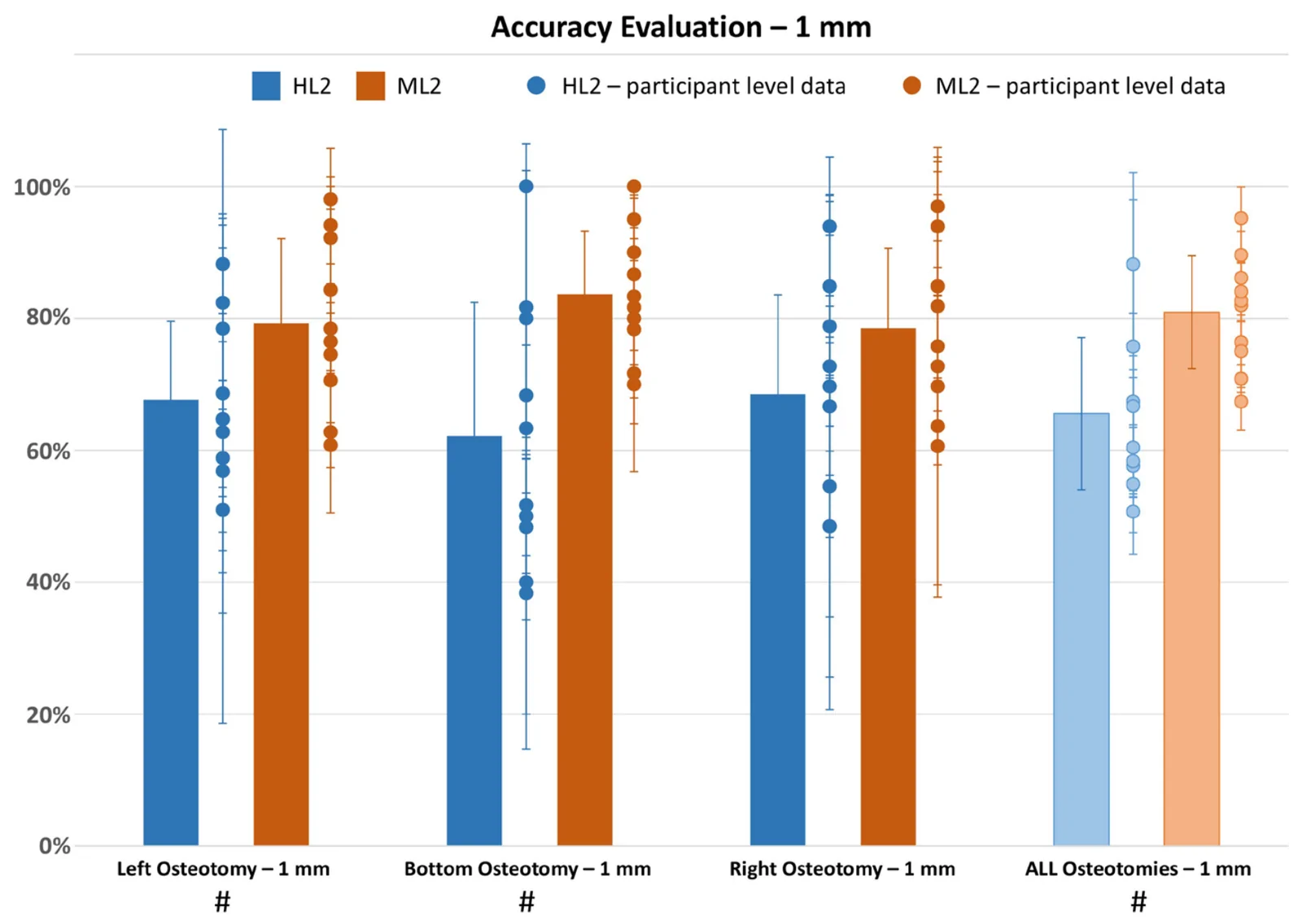
Accuracy comparison of HL2 and ML2 in AR-assisted tracing of planned osteotomies for oncological resection, at 1 mm threshold. Circles represent participant-level paired data; bars represent the average accuracy. # indicates statistically significant differences. The bars in light colors represent accuracy comparison for all osteotomies (i.e. left, bottom, right).

At the 1 mm threshold (Figure 6), ML2 generally showed higher accuracy values than HL2 across the evaluated osteotomy sites and in the pooled analysis. Following Bonferroni correction (*p* < 0.0167), significant differences were observed for the left and bottom osteotomies and in the overall analysis, while the right osteotomy did not show a statistically significant difference (Table 3). Overall, these results indicate that ML2 was associated with higher accuracy at the 1 mm threshold in most comparisons.

**Figure 7:**
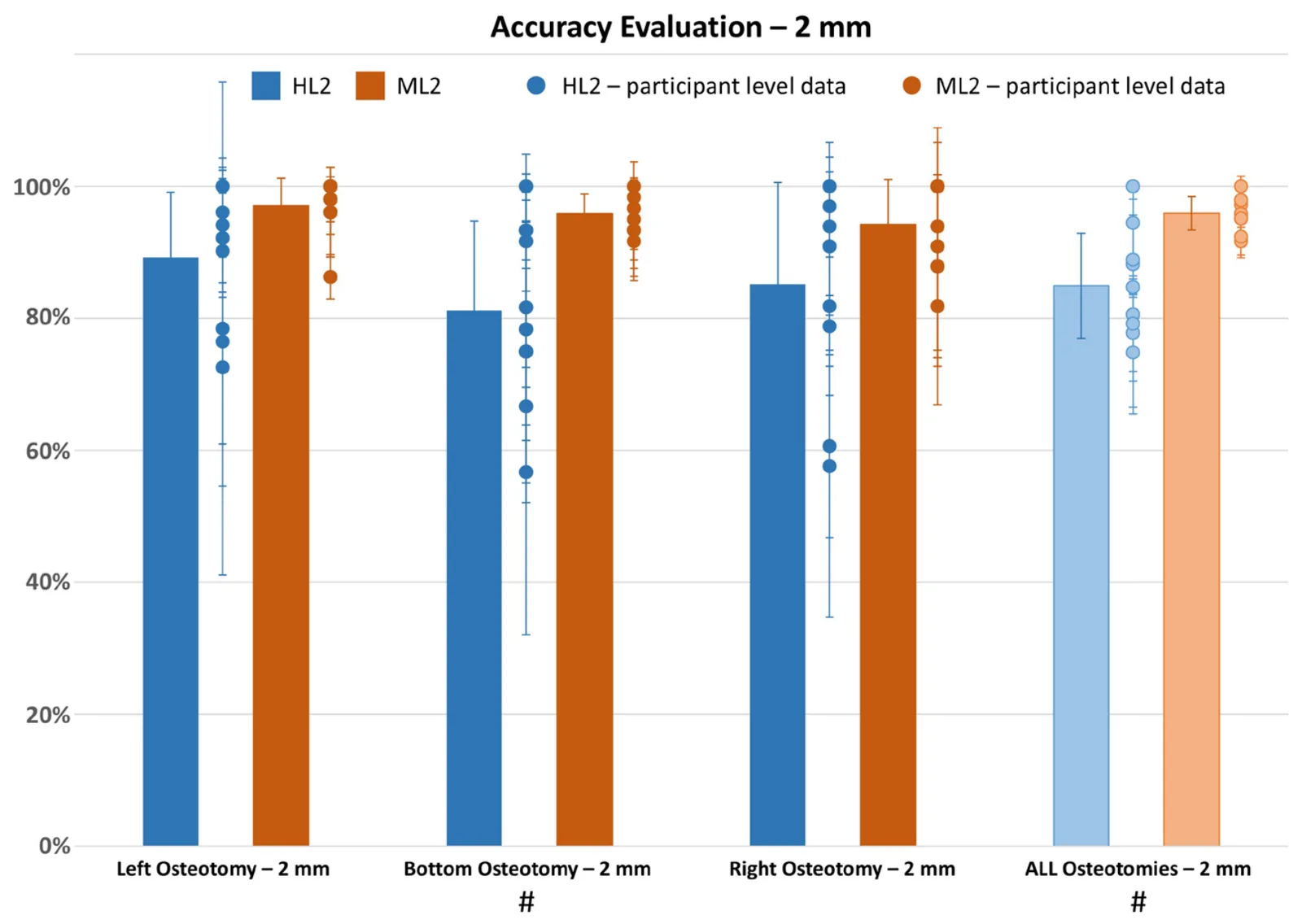
Accuracy comparison of HL2 and ML2 in AR-assisted tracing of planned osteotomies for oncological resection, at 2 mm threshold. Circles represent participant-level paired data; bars represent the average accuracy. # indicates statistically significant differences. The bars in light colors represent accuracy comparison for all osteotomies (i.e. left, bottom, right).

At the 2 mm threshold (Figure 7), both devices demonstrated improved performance compared with the 1 mm threshold. ML2 consistently showed higher accuracy values than HL2 across all osteotomy sites and in the pooled analysis. According to the Bonferroni-corrected results reported in Table 3 (*p* < 0.0167), statistically significant differences were found only for the bottom osteotomy and the overall analysis, while the left and right osteotomies did not show significant differences.

**Figure 8:**
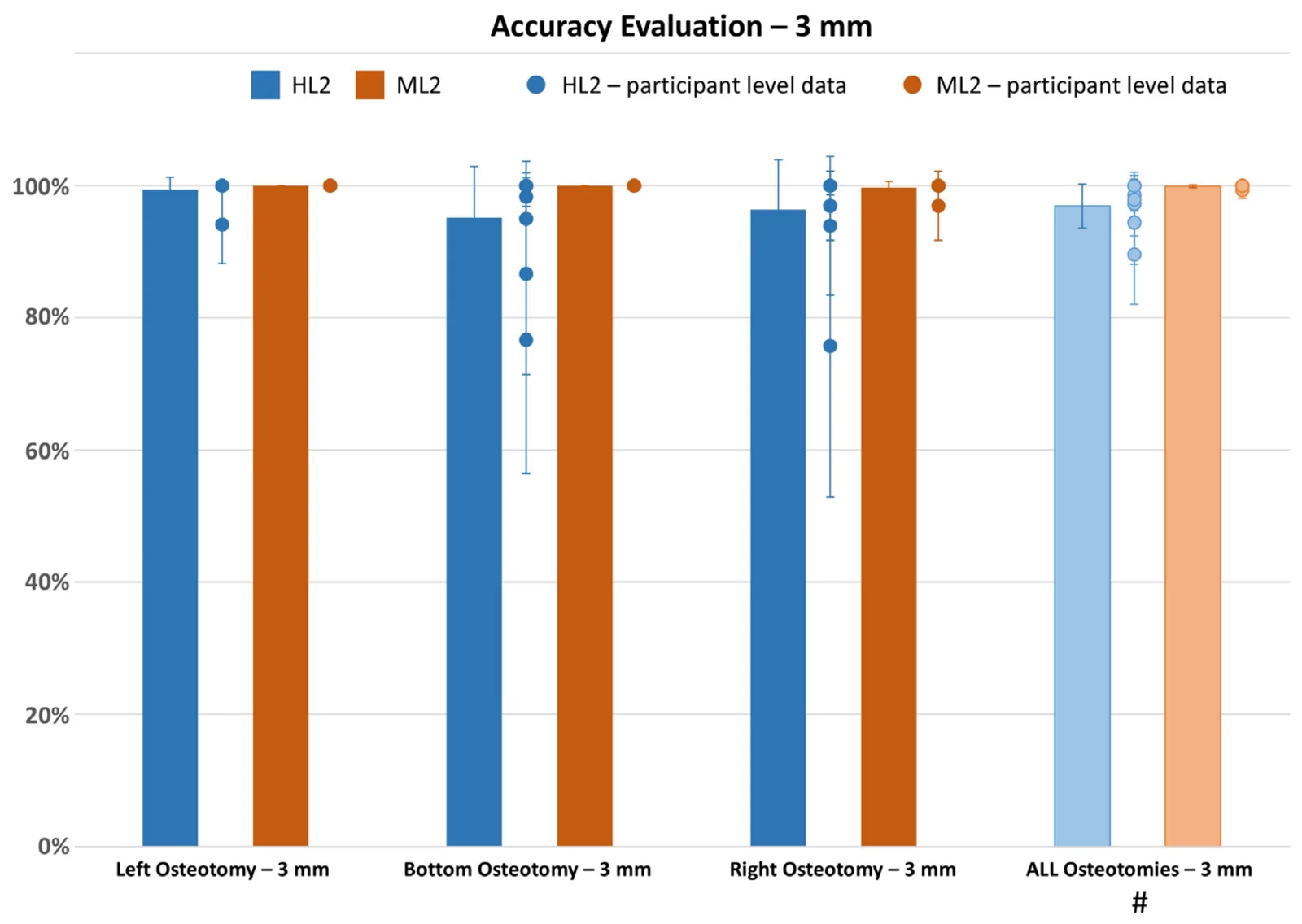
Accuracy comparison of HL2 and ML2 in AR-assisted tracing of planned osteotomies for oncological resection, at 3 mm threshold. Circles represent participant-level paired data; bars represent the average accuracy. # indicates statistically significant differences. The bars in light colors represent accuracy comparison for all osteotomies (i.e. left, bottom, right).

At the 3 mm threshold (Figure 8), both HL2 and ML2 achieved near-optimal accuracy, with performance consistently approaching or exceeding 95–100% across all osteotomies.

The difference between the two devices narrowed considerably, reflecting convergence in performance at larger thresholds.

Although ML2 continued to show slightly higher accuracy values, most differences were no longer statistically significant. As reported in Table 3, after Bonferroni correction (*p* < 0.0167), statistical significance was retained only in the overall pooled analysis, while no significant differences were found for the individual osteotomy sites.

Details on exact *p*-values, effect sizes, and confidence intervals obtained for each comparison are reported in Table 3.

**Figure 9:**
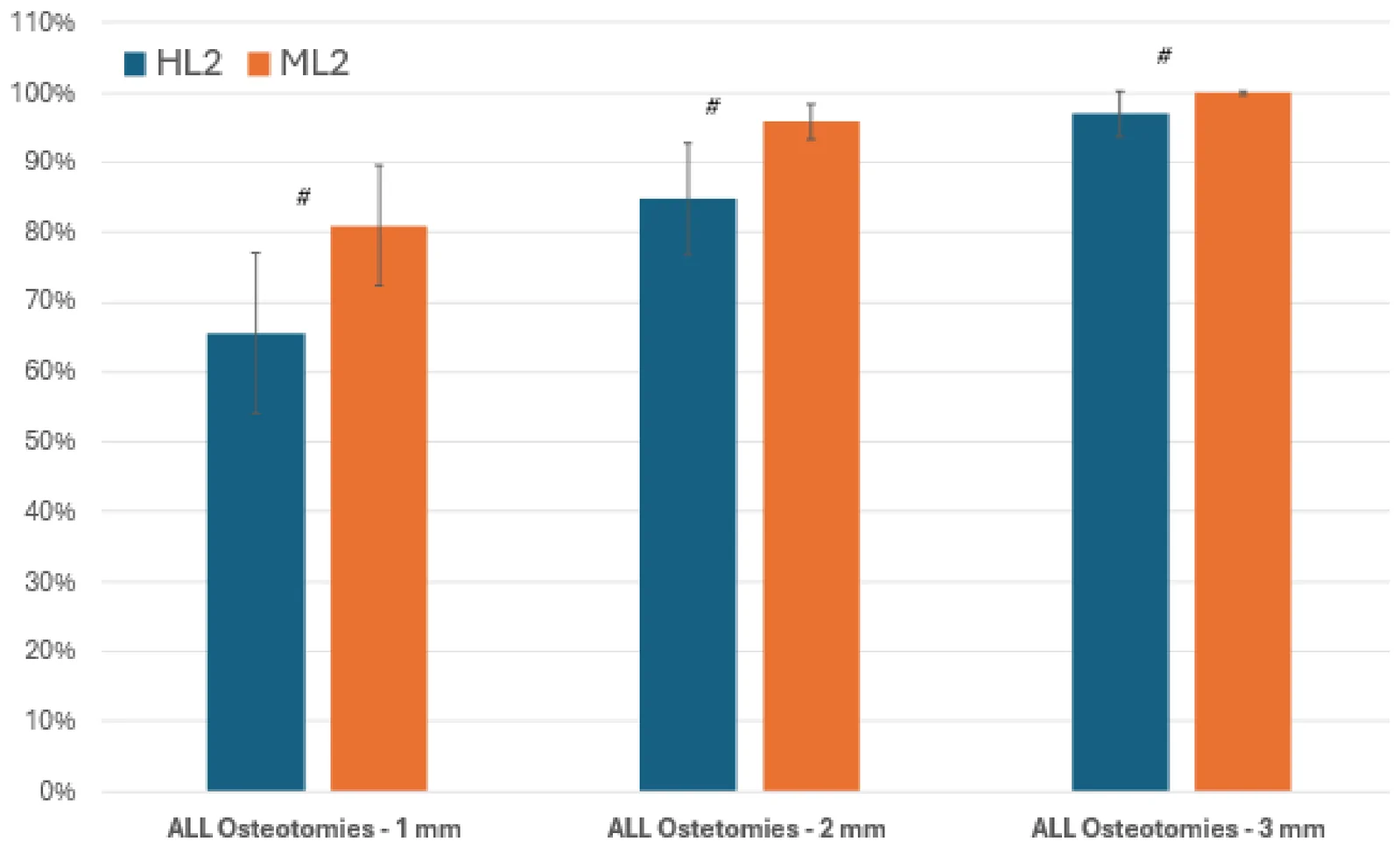
Accuracy comparison of HL2 and ML2 in AR-assisted tracing of planned osteotomies for oncological resection, at all thresholds (1 mm, 2 mm and 3 mm). # indicates a statistically significant difference.

Overall, ML2 showed higher accuracy than HL2 under the specific experimental conditions tested, particularly at narrower margin thresholds; however, these findings should be considered exploratory (Figure 9). As the accuracy threshold increased, the performance of both devices improved, with differences between them almost diminishing at the 3 mm threshold. These findings emphasized the clinical relevance of AR guidance, especially in scenarios requiring maximal preservation of healthy tissue and precise resection margins.

### 3.2. Technical Differences

Several technical differences exist between ML2 and HL2 that may be relevant when considering their use in surgical environments (Table 1). As a newer-generation device, ML2 incorporates more advanced hardware, greater processing capabilities, and improved display performance compared with HL2. It also offers a wider field of view, higher refresh rates, multiple focal planes, and enhanced SLAM functionality designed for dynamic environments. Both devices support eye, hand, and voice tracking, although their implementation differs. From an ergonomic perspective, ML2 is considerably lighter than HL2, which may improve user comfort during extended procedures, while its external compute pack may introduce practical considerations in the operating room. Overall, these technical differences provide useful context for interpreting the study findings; however, the present study was not designed to determine the specific contribution of individual device characteristics to the observed performance outcomes.

### 3.3. Ergonomics Differences

ML2 exhibited enhanced ergonomic performance across all metrics in comparison to HL2. Regarding adjustability and fit, ML2 received an average score of 4.0, surpassing HL2’s score of 3.3, signifying superior comfort for extended use. Comfort during clinical tasks and device weight received higher ratings for ML2 (3.4 and 4.0, respectively) compared to HL2 (3.1 and 3.2), underscoring enhanced balance and less fatigue.

Participants reported higher scores for hologram quality and integration with ML2 (3.8 vs. 2.8), indicating perceived differences in the visualization experience between the two devices.

Likewise, engagement with holograms (4.0 vs. 3.1) and reaction velocity (4.1 vs. 2.9) were higher for ML2, indicating a trend toward a more responsive interaction experience.

The hand identification feature in ML2 was enhanced (3.0 vs. 2.3); however both systems exhibited possibilities for further optimisation. The FOV was wider and more comfortable in ML2 (3.9 compared to 3.3), aligning with its expanded optical projection area. Finally, the assessment of fatigability with continuous use was marginally superior for ML2 (4.5 compared to 4.1), suggesting less strain during longer tasks with both devices.

Participants reported a preference for ML2 in terms of visual quality, responsiveness, and extended usability. These perceived advantages may contribute to its suitability for surgical and clinical AR applications, although further investigation in real-world settings is warranted.

**Figure 10:**
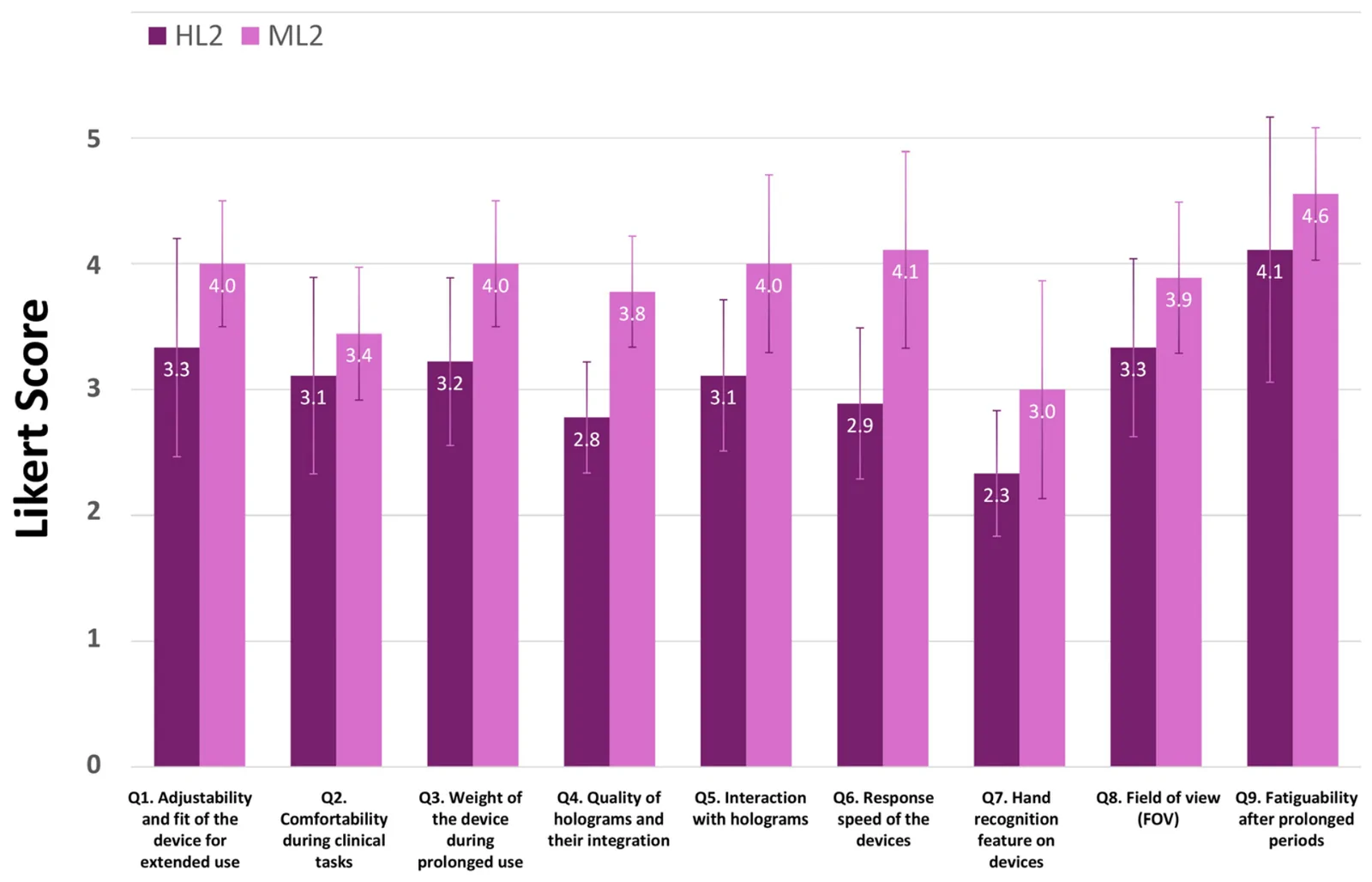
Representation of the survey results using a Likert scale.

Exploratory descriptive results from the in-house ergonomics questionnaire are presented in Figure 10 as mean values and standard deviations. No inferential statistical comparisons were performed for these data.

## 4. Discussion

This study conducted a direct comparative assessment of two advanced AR HMDs (HL2 and ML2), within the realm of orthopedic oncology, utilizing a patient-specific pelvic bone phantom as a controlled experimental model. The results indicate that both devices can effectively enable precise AR-assisted tracing of planned osteotomies; however, ML2 consistently surpassed HL2 in precision, visualization quality, and user ergonomics, indicating its potential to support procedures demanding high levels of accuracy.

The quantitative analysis revealed differences in accuracy between ML2 and HL2 across the evaluated osteotomy locations and margin thresholds. At the 1 mm and 2 mm thresholds, higher accuracy values were observed with ML2 (85–95%) than with HL2 (65–85%). Some comparisons remained statistically significant after Bonferroni correction for multiple comparisons. These findings provide preliminary evidence of differences between the two devices under the specific experimental conditions tested.

At the 3 mm threshold, both devices exhibited near-optimal accuracy (95–100%), with the disparity between them significantly diminishing. This convergence suggests that while both devices may be adequate for larger oncologic margins, the trend toward greater accuracy of ML2 becomes particularly evident under high-precision conditions, such as limb-sparing resections or pelvic tumor excisions, where millimeter-level accuracy is critical.

These findings validate other studies highlighting the clinical efficacy of AR in enhancing spatial orientation and intraoperative confidence during bone tumor resections. The trend toward higher accuracy observed with ML2 may be related to several hardware and software characteristics, including its wider field of view, higher display refresh rate, and different depth perception mechanisms. However, the present study was not designed to isolate the contribution of these individual factors. Therefore, any association between device features and performance outcomes should be interpreted with caution.

The comparison of the technical specifications of the two headsets may provide possible explanations for the observed performance differences. ML2 incorporates a more powerful computing architecture and greater memory capacity than HL2, features that could potentially support more demanding rendering and tracking tasks. Similarly, the increased RAM and storage capacity (16 GB/256 GB compared to 4 GB/64 GB) may facilitate the management of larger datasets and higher fidelity 3D models, which are essential for executing complex pelvic reconstructions. Nevertheless, the present study did not directly evaluate the impact of these technical characteristics on performance.

The optical system may also have influenced user experience and task performance. ML2 incorporates a multi-focal plane waveguide display and adaptive dimming zones which have been proposed to reduce the vergence-accommodation conflict, compared with fixed focal-depth displays, such as that of HoloLens 2, which has a fixed focal depth of approximately 2 m. This may yield a more authentic depth perception of holograms, enabling surgeons to sustain accurate alignment between the virtual and physical anatomies. The ML2 necessitates an external compute pack, potentially raising minor sterility issues in clinical settings; nonetheless, this design choice significantly reduces the headset weight (260 g compared to 566 g), enhancing comfort during extended surgical procedures.

The ergonomic assessment also revealed a distinct user preference for the ML2. Participants assigned higher ratings to ML2 across all nine assessed categories on the Likert scale, encompassing comfort, fit, visual clarity, and interaction responsiveness. The enhanced adjustability, less fatigue, and exceptional holographic quality noted by users align with its sophisticated optical and mechanical build. The wider FOV and improved hand tracking capabilities may have contributed to an increase in user immersion and work efficiency, factors essential for clinical adoption where cognitive load and situational awareness are critical.

Notably, although ML2 offers enhanced comfort, certain users emphasized the practical difficulty associated with its external computing pack, indicating that while it has technical benefits, its incorporation into sterile environments necessitates further adjustments to workflow. Overall feedback suggests that ML2 provides a more sophisticated and flexible user experience, enhancing sustained focus during extended tasks. Conversely, many users encountered displacement of holograms during surgical procedures with HL2, and some also observed the flickering of superimposed holograms. Several individuals expressed dissatisfaction with HL2’s slow speed, slow registration process, and hand motion detection compared to ML2.

From a clinical perspective, our findings suggest that AR technologies may support visualization and spatial understanding during complex orthopedic oncology procedures. The performance observed with ML2 in this experimental setting indicates its potential utility for tasks requiring precise anatomical identification particularly in scenarios where the preservation of vital neurovascular and muscular components is crucial. Improved precision may help decrease positive resection rates and enhance functional results, thus addressing an important constraint in musculoskeletal oncology. However, the findings derive from a single patient-specific phantom and should not be interpreted as evidence of superior clinical performance or improved surgical outcomes.

The patient-specific phantom approach used in this study may represent a useful framework for future comparative evaluations of AR platforms and their integration with surgical navigation technologies. Further validation across multiple anatomical scenarios will be required to confirm its broader applicability.

### Limitations

This work offers extensive insights, although certain limitations must be recognised. The experiment was initially performed on in-vitro phantoms, which, although anatomically accurate, fail to recreate all intraoperative variables, including tissue deformation, hemorrhage, and post-operative functional outcomes. Furthermore, translating marker-based tracking to in-vivo settings may present additional challenges, particularly regarding the proper fitting and stability of the tracking template, which can be more critical in a real surgical environment. Potential issues related to variable lighting conditions in the operating room may also affect the visibility and accuracy of AR overlays, potentially impacting performance during real procedures. As this feasibility study was designed to provide an initial comparison of the two AR platforms in a challenging orthopedic oncology scenario, future investigations involving multiple anatomical models and clinical cases will be required to establish the generalizability of the findings and support broader conclusions regarding platform performance. Also, the limited sample size (*n* = 10) and the absence of a priori power analysis restrict the statistical power and generalizability of the findings. Therefore, the observed differences between devices should be considered preliminary and interpreted only within the specific experimental conditions investigated. Larger, adequately powered studies are needed to confirm these findings.

A limitation of this study is that participants performed a line-tracing task on a patient-specific phantom rather than a true osteotomy. Therefore, the experimental setup did not account for factors inherent to surgical bone cutting, including saw handling, blade orientation, kerf width, tool deflection, haptic feedback, and bone-tissue interactions. Consequently, the observed performance should be interpreted as reflecting AR-guided visualization and tracing accuracy rather than actual osteotomy execution.

Accuracy assessment was based on predefined tolerance thresholds rather than continuous geometric deviation measurements. Consequently, the proposed methodology provides a categorical evaluation of performance but does not capture the full extent of deviations from the planned osteotomy trajectory. Although this approach enabled a preliminary characterization of tracing performance at different deviation levels, it does not provide the granularity of a continuous quantitative error metric and limits the clinical interpretation of the results. Future studies should therefore quantify spatial deviation continuously and report corresponding measures of variability and uncertainty.

To provide a broader context for the accuracy observed in our experimental setting, although direct comparisons with freehand techniques or surgical navigation systems were not performed, previous studies in orthopedic oncology have reported millimeter-scale errors for conventional approaches, including approximately 3 ± 1 mm for freehand and 2 ± 0 mm for navigation-assisted procedures in a preclinical model [21,22].

Thus, the accuracy observed in the present AR-based experiments appears to be within the order of magnitude reported for established guidance approaches, although dedicated head-to-head studies are required to establish equivalence or potential added value.

Moreover, although all the 3D-printed accuracy templates were visually inspected and verified for proper fit on the corresponding anatomical model before testing, dimensional deviations introduced during the manufacturing process were not quantified and may have influenced the reported accuracy measurements.

Another limitation is that the reported tracing error represents the combined effect of multiple sources of error within the AR workflow, including tracking, registration, visualization, and user interaction. The technical performance of the tracking systems was not quantitatively characterized in terms of registration error, jitter, drift, or latency. Consequently, the individual contribution of each component could not be independently quantified. Future studies should incorporate dedicated technical measurements of tracking and registration performance alongside task-level accuracy assessment.

A further limitation is that the application rendering frame rate was not quantitatively monitored during the experiments. While no perceptible performance issues were observed, future studies should include objective performance metrics, such as frame rate, latency, and tracking stability measurements, to further characterize the technical performance of the AR system.

Ergonomics and usability assessment relied on an in-house, study-specific questionnaire administered to a limited number of participants, rather than a standardized and validated instrument. Consequently, the findings related to user experience and ergonomics should be considered exploratory and cannot be interpreted as a validated assessment of usability or ergonomic performance. Future investigations should integrate validated measures such as the NASA-TLX, SUS, or USE questionnaires to provide a more comprehensive and comparable assessment of usability and cognitive workload, particularly under the high-stress, time-sensitive conditions of the operating room, where surgical demands and decision-making pressures may substantially influence the usability and clinical integration of AR systems.

Finally, although the order of headset use was counterbalanced across participants, participant allocation was not randomized. Therefore, a residual learning effect cannot be completely excluded.

## 5. Conclusions

Differences between ML2 and HL2 were observed in terms of accuracy and exploratory ergonomics and user-experience outcomes, with an overall trend favoring ML2 under the specific experimental conditions evaluated.

The combination of a larger field of view, improved optical clarity, lightweight design, and advanced processing capabilities may contribute to ML2’s suitability for AR-assisted orthopedic applications. However, given the pilot nature of the study, the absence of comparisons with conventional navigation systems or freehand technique, and the lack of continuous deviation measurements, these findings should be interpreted with caution. Further studies involving larger cohorts, clinically relevant scenarios, and comparisons with established navigation techniques are required to confirm the potential advantages of ML 2 for precision-guided bone tumor resections and other orthopedic procedures. Although the observed improvements in tracing accuracy are encouraging, their potential impact on oncological and functional outcomes remains to be established through dedicated clinical studies.

## Figures and Tables

**Table 1:** Technical differences between Magic Leap 2 and HoloLens 2.

	Magic Leap 2	HoloLens 2
**Release Date**	September 2022	November 2019
**Processor**	AMD Zen 2 Quad-core + Custom Co-processors	Qualcomm Snapdragon 850 + Custom HPU 2.0
**GPU**	AMD RDNA 2 @ 1.1 GHz, 1SA, 4WGP (8 CU), 2RB+, 1MB L2 Cache	Integrated Adreno 630
**OS/Platform**	Lumin OS (Android-based custom OS)	Windows Holographic (Windows 10-based)
**Display Type**	Waveguide with LCoS displays	Waveguide with laser-based MEMS
**Resolution**	1440 × 1760 per-eye	1440 × 936 per-eye
**Field of View (FOV)**	70° diagonal field of view	52° diagonal field of view
**Refresh Rate**	Up to 120 Hz	60 Hz
**Eye Tracking**	Yes	Yes
**Hand Tracking**	Yes	Yes
**Voice commands**	Yes (custom voice commands)	Yes (Microsoft Cortana)
**Sensors**	IMU, depth, ambient light, world cameras, eye tracking	IMU, depth sensor, IR, ambient light sensor
**Focal Planes**	Multiple focal planes (dynamic dimming zones): default setting of 37 cm (adjustable to 25 cm minimum) to infinity	Single fixed focal plane (2 m)
**SLAM (Simultaneous Localization and Mapping)**	Yes—Enhanced (optimized for dynamic environments)	Yes—Advanced (via sensors + HPU 2.0)
**RAM/Storage**	16GB LPDDR5 5500/256 GB	4 GB LPDDR4X/64 GB
**Weight**	260 g headset (with external compute pack)	566 g (fully integrated)
**Battery life**	3.5 h typical use (7 h in sleep mode)	2–3 h of active use
**Developer SDK**	Magic Leap SDK, Unity, Unreal	MRTK, Unity, Unreal
**Price (Launch)**	$3299	$3500

**Table 2:** Survey Comparing HoloLens 2 and Magic Leap 2 for Clinical Applications.

Section	Question	Answer	
**General Information**	Have you had any prior experience with augmented reality (AR) vision systems in general?	Yes/No	
	Have you used either HoloLens 2 or Magic Leap 2 (or both) for clinical applications?	Yes/No	
		**HoloLens 2**	**Magic Leap 2**
**Comfort and Wearability**	1. How would you rate the adjustability and fit of the device for extended use?	- Poor - Fair - Good - Very Good - Excellent	- Poor - Fair - Good - Very Good - Excellent
	2. How comfortable is the device to wear during clinical tasks?	- Poor - Fair - Good - Very Good - Excellent	- Poor - Fair - Good - Very Good - Excellent
	3. How would you rate the weight of the device on your head during prolonged use?	- Too Heavy - Heavy - Slightly Heavy - Just Right - Lightweight	- Too Heavy - Heavy - Slightly Heavy - Just Right - Lightweight
**Hologram Quality and Interaction**	4. How would you rate the quality of holograms and their integration into the real world?	- Poor - Fair - Good - Very Good - Excellent	- Poor - Fair - Good - Very Good - Excellent
	5. How intuitive and realistic is the interaction with holograms?	- Not Intuitive - Slightly Intuitive - Moderately Intuitive - Very Intuitive - Extremely Intuitive	- Not Intuitive - Slightly Intuitive - Moderately Intuitive - Very Intuitive - Extremely Intuitive
	6. How would you rate the response speed of the device during interactions?	- Very Slow - Slow - Moderate - Fast - Very Fast	- Very Slow - Slow - Moderate - Fast - Very Fast
**Recognition and Field of View**	7. How accurate and reliable is the hand recognition feature on devices?	- Poor - Fair - Good - Very Good - Excellent	- Poor - Fair - Good - Very Good - Excellent
	8. How would you rate the field of view (FOV) on both devices?	- Too Narrow - Slightly Narrow - Adequate - Wide - Very Wide	- Too Narrow - Slightly Narrow - Adequate - Wide - Very Wide
**Fatiguability**	9. Have you experienced fatigue after wearing the device for extended periods?	- Total fatigue/ Exhaustion - Very fatigued - Moderate Fatigue - Little fatigue - No fatigue	- Total fatigue/ Exhaustion - Very fatigued - Moderate Fatigue - Little fatigue - No fatigue
**Additional Feedback**	Do you have any additional comments or observations about the HoloLens 2 or Magic Leap 2 in clinical settings?	Open-ended response	Open-ended response

**Table 3:** Detailed statistical results for the accuracy comparison for HL2 and ML2 at the three thresholds. # indicates statistically significant differences according to Bonferroni correction.

	*p*	(ML2-HL2) % Accuracy				Effect Size	
Experimental Condition		Mean	SD	95% CI		Cohen’s d	Description
**Left Osteotomy—1 mm**	0.0113 (#)	12%	13%	2%	21%	0.87	Large
**Bottom Osteotomy—1 mm**	0.0010 (#)	22%	16%	10%	33%	1.36	Very Large
**Right Osteotomy—1 mm**	0.0416	10%	16%	−2%	22%	0.62	Medium
**All—1 mm**	0.0002 (#)	15%	9%	9%	21%	1.79	Very Large
**Left Osteotomy—2 mm**	0.0268	8%	11%	0%	16%	0.70	Medium
**Bottom Osteotomy—2 mm**	0.0022 (#)	15%	12%	6%	23%	1.19	Large
**Right Osteotomy—2 mm**	0.0273	9%	13%	0%	18%	0.70	Medium
**All—2 mm**	0.0002 (#)	11%	7%	6%	16%	1.68	Very Large
**Left Osteotomy—3 mm**	0.1717	1%	2%	−1%	2%	0.32	Small
**Bottom Osteotomy—3 mm**	0.0401	5%	8%	−1%	10%	0.62	Medium
**Right Osteotomy—3 mm**	0.0963	3%	7%	−2%	9%	0.45	Small
**All—3 mm**	0.0101 (#)	3%	3%	1%	5%	0.89	Large

## Data Availability

The raw data supporting the conclusions of this article will be made available by the authors upon reasonable request.

## References

[1] Zari G., Condino S., Cutolo F., Ferrari V. (2023). Magic Leap 1 versus Microsoft HoloLens 2 for the Visualization of 3D Content Obtained from Radiological Images. Sensors.

[2] Pose-Díez-de-la-Lastra A., Moreta-Martinez R., García-Sevilla M., García-Mato D., Calvo-Haro J.A., Mediavilla-Santos L., Pérez-Mañanes R., von Haxthausen F., Pascau J. (2022). HoloLens 1 vs. HoloLens 2: Improvements in the New Model for Orthopedic Oncological Interventions. Sensors.

[3] Cannizzaro D., Zaed I., Safa A., Jelmoni A.J.M., Composto A., Bisoglio A., Schmeizer K., Becker A.C., Pizzi A., Cardia A. (2022). Augmented Reality in Neurosurgery, State of Art and Future Projections. A Systematic Review. Front. Surg..

[4] Roberts S., Desai A., Checcucci E., Puliatti S., Taratkin M., Kowalewski K.-F., Rivas J.G., Rivero I., Veneziano D., Autorino R. (2022). “Augmented reality” applications in urology: A systematic review. Minerva Urol. Nephrol..

[5] Bianchi L., Chessa F., Angiolini A., Cercenelli L., Lodi S., Bortolani B., Molinaroli E., Casablanca C., Droghetti M., Gaudiano C. (2021). The Use of Augmented Reality to Guide the Intraoperative Frozen Section During Robot-assisted Radical Prostatectomy. Eur. Urol..

[6] Schiavina R., Bianchi L., Chessa F., Barbaresi U., Cercenelli L., Lodi S., Gaudiano C., Bortolani B., Angiolini A., Bianchi F.M. (2021). Augmented Reality to Guide Selective Clamping and Tumor Dissection During Robot-assisted Partial Nephrectomy: A Preliminary Experience. Clin. Genitourin. Cancer.

[7] Rad A.A., Vardanyan R., Lopuszko A., Alt C., Stoffels I., Schmack B., Ruhparwar A., Zhigalov K., Zubarevich A., Weymann A. (2022). Virtual and Augmented Reality in Cardiac Surgery. Braz. J. Cardiovasc. Surg..

[8] Molina C.A., Dibble C.F., Lo S.L., Witham T., Sciubba D.M. (2021). Augmented reality-mediated stereotactic navigation for execution of en bloc lumbar spondylectomy osteotomies. J. Neurosurg. Spine.

[9] Gouveia P.F., Costa J., Morgado P., Kates R., Pinto D., Mavioso C., Anacleto J., Martinho M., Lopes D.S., Ferreira A.R. (2021). Breast cancer surgery with augmented reality. Breast.

[10] Mahmud N., Cohen J., Tsourides K., Berzin T. (2015). Computer vision and augmented reality in gastrointestinal endoscopy. Gastroenterol. Rep..

[11] Golse N., Petit A., Lewin M., Vibert E., Cotin S. (2021). Augmented Reality during Open Liver Surgery Using a Markerless Non-rigid Registration System. J. Gastrointest. Surg..

[12] Nasir N., Cercenelli L., Tarsitano A., Marcelli E. (2023). Augmented reality for orthopedic and maxillofacial oncological surgery: A systematic review focusing on both clinical and technical aspects. Front. Bioeng. Biotechnol..

[13] Ruggiero F., Cercenelli L., Tarsitano A., Emiliani N., Stradiotti S., Babini M., Gardenghi B., Lefosse M., Marcelli E., Zucchelli M. (2025). Augmented reality in pediatric craniofacial surgery: Clinical experience. J. Neurosurg. Pediatr..

[14] Cercenelli L., Emiliani N., Gulotta C., Bevini M., Badiali G., Marcelli E. (2025). Augmented Reality to Assist in the Diagnosis of Temporomandibular Joint Alterations. Inf. Syst. Front..

[15] Puleio F., Tosco V., Pirri R., Simeone M., Monterubbianesi R., Giudice G.L., Giudice R.L. (2024). Augmented Reality in Dentistry: Enhancing Precision in Clinical Procedures—A Systematic Review. Clin. Pract..

[16] Bertrand T.E., Cruz A., Binitie O., Cheong D., Letson D.G. (2016). Do Surgical Margins Affect Local Recurrence and Survival in Extremity, Nonmetastatic, High-grade Osteosarcoma?. Clin. Orthop. Relat. Res..

[17] García-Sevilla M., Moreta-Martinez R., García-Mato D., Pose-Diez-De-La-Lastra A., Pérez-Mañanes R., Calvo-Haro J.A., Pascau J. (2021). Augmented Reality as a Tool to Guide PSI Placement in Pelvic Tumor Resections. Sensors.

[18] Moreta-Martinez R., García-Mato D., García-Sevilla M., Pérez-Mañanes R., Calvo-Haro J., Pascau J. (2018). Augmented reality in computer-assisted interventions based on patient-specific 3D printed reference. Healthc. Technol. Lett..

[19] Ceccariglia F., Cercenelli L., Badiali G., Marcelli E., Tarsitano A. (2022). Application of Augmented Reality to Maxillary Resections: A Three-Dimensional Approach to Maxillofacial Oncologic Surgery. J. Pers. Med..

[20] Yang Z., Su X., Yuan Z., Yuan Y., Liu J., Wang Z., Jin K., Ye N., Zhang J., Cai J. (2025). Mixed reality holographic navigation for intracranial lesions using HoloLens 2: A pilot study and literature review. Clin. Neurol. Neurosurg..

[21] Bosma S.E., Wong K.C., Paul L., Gerbers J.G., Jutte P.C. (2018). A Cadaveric Comparative Study on the Surgical Accuracy of Freehand, Computer Navigation, and Patient-Specific Instruments in Joint-Preserving Bone Tumor Resections. Sarcoma.

[22] Chan H.H.L., Nayak P., Alshaygy I., Gundle K.R., Tsoi K.M., Daly M.J., Irish J.C.M., Ferguson P.C.M., Wunder J.S.M. (2024). Does Freehand, Patient-specific Instrumentation or Surgical Navigation Perform Better for Allograft Reconstruction After Tumor Resection? A Preclinical Synthetic Bone Study. Clin. Orthop. Relat. Res..

